# Race and Ethnicity Misclassification in Hospital Discharge Data and the Impact on Differences in Severe Maternal Morbidity Rates in Florida

**DOI:** 10.3390/ijerph20095689

**Published:** 2023-04-30

**Authors:** Chinyere N. Reid, Renice Obure, Jason L. Salemi, Chinwendu Ilonzo, Judette Louis, Estefania Rubio, William M. Sappenfield

**Affiliations:** 1 Chiles Center, College of Public Health, University of South Florida, Tampa, FL 33612, USA; 2 Department of Obstetrics & Gynecology, Morsani College of Medicine, University of South Florida, Tampa, FL 33612, USA

**Keywords:** race and ethnicity, misclassification, severe maternal morbidity, hospital discharge, birth certificate

## Abstract

Hospital discharge (HD) records contain important information that is used in public health and health care sectors. It is becoming increasingly common to rely mostly or exclusively on HD data to assess and monitor severe maternal morbidity (SMM) overall and by sociodemographic characteristics, including race and ethnicity. Limited studies have validated race and ethnicity in HD or provided estimates on the impact of assessing health differences in maternity populations. This study aims to determine the differences in race and ethnicity reporting between HD and birth certificate (BC) data for maternity hospitals in Florida and to estimate the impact of race and ethnicity misclassification on state- and hospital-specific SMM rates. We conducted a population-based retrospective study of live births using linked BC and HD records from 2016 to 2019 (*n* = 783,753). BC data were used as the gold standard. Race and ethnicity were categorized as non-Hispanic (NH)-White, NH-Black, Hispanic, NH-Asian Pacific Islander (API), and NH-American Indian or Alaskan Native (AIAN). Overall, race and ethnicity misclassification and its impact on SMM at the state- and hospital levels were estimated. At the state level, NH-AIAN women were the most misclassified (sensitivity: 28.2%; positive predictive value (PPV): 25.2%) and were commonly classified as NH-API (30.3%) in HD records. NH-API women were the next most misclassified (sensitivity: 57.3%; PPV: 85.4%) and were commonly classified as NH-White (5.8%) or NH-other (5.5%). At the hospital level, wide variation in sensitivity and PPV with negative skewing was identified, particularly for NH-White, Hispanic, and NH-API women. Misclassification did not result in large differences in SMM rates at the state level for all race and ethnicity categories except for NH-AIAN women (% difference 78.7). However, at the hospital level, Hispanic women had wide variability of a percent difference in SMM rates and were more likely to have underestimated SMM rates. Reducing race and ethnicity misclassification on HD records is key in assessing and addressing SMM differences and better informing surveillance, research, and quality improvement efforts.

## 1. Introduction

Severe maternal morbidity (SMM) is an unexpected life-threatening complication of childbirth in which women suffer short-term or long-term health consequences if not identified and treated in a timely manner [1]. In the United States (USA), SMM is identified using 21 indicator conditions, and the five most common conditions are blood transfusion, disseminated intravascular coagulation, hysterectomy, acute renal failure, and adult respiratory distress syndrome [2]. From 2011 to 2020, SMM rates have increased in the USA from 69.8 to 88.2 women per 10,000 hospital deliveries, and in the state of Florida, USA, from 68.6 to 94.3 women per 10,000 hospital deliveries [3]. The literature suggests that increases in risk factors such as maternal age, prepregnancy obesity, pre-existing chronic conditions, and cesarean delivery may be responsible for the increase in SMM rates [4].

SMM mostly affects women who identify as racial and ethnic minorities [5,6], with most of the focus on assessing these disparities being placed on the influence of social determinants of health such as poverty, education, neighborhood characteristics, etc. [7,8]. The literature supports that the association between race and ethnicity and SMM remains even after accounting for sociodemographic factors, pregnancy factors, and health system factors [9,10,11]. However, race and ethnicity differences in SMM are complex, and little is known as to how misclassification of race and ethnicity may influence these disparities. When compared to non-Hispanic White (NH-White) women, a multistate analysis reported SMM rates that were 2.1, 1.3, 1.2, and 1.7 times higher for non-Hispanic Black (NH-Black), Hispanic, Asian Pacific Islander (API), and American Indian Alaska Native (AIAN), respectively [12]. Accurate race and ethnicity documentation are essential to quality improvement, SMM prevention, and reduction in health inequities. Misclassification of race and ethnicity can result in incorrect estimation of the burden of health outcomes [13]. It is becoming increasingly common for researchers, public health, and hospitals to rely mostly or exclusively on hospital discharge (HD) data to assess and monitor SMM overall and by various sociodemographic characteristics, including race and/or ethnicity. This is problematic because studies consistently report misclassification of NH-API and NH-AIAN race and ethnicity groups in non-maternity populations [14,15,16,17]. It is less clear the extent to which misclassification impacts maternity populations.

The study objectives were as follows: (1) determining the differences in race and ethnicity reporting between HD and birth certificate (BC) data from maternity hospitals in Florida and (2) estimating the impact of race and ethnicity misclassification on state- and hospital-specific SMM rates.

## 2. Materials and Methods

### 2.1. Data Sources

We used the latest available data to conduct a population-based retrospective study using Florida BC linked to inpatient HD records for live births from January 2016 to December 2019. The study time period was chosen because 2016 was the first full year of HD data following the ICD-10-CM implementation. BC records were provided by the Florida Department of Health (FDOH, Tallahassee, FL, USA) and contain demographic and clinical information on the mother and baby. HD records were provided by the Florida Agency for Health Care Administration (AHCA, Tallahassee, FL, USA) and contain individual-level patient data for each inpatient hospital stay. Hospitals are required to submit race and ethnicity in a standard format to AHCA. However, how this information is actually collected varies across hospitals and may be self-reported by patients or designated from observation by admitting clerks or clinical staff [18,19,20,21]. A hierarchical, deterministic data linkage algorithm performed by the FDOH was used to link BC and HD records; this linkage process has been described elsewhere [22,23,24]. Among non-federal maternity hospitals in the state, the linkage rate was 91.3%. Home births and births at military hospitals were excluded since these data were not captured in the data from AHCA. This study was reviewed and approved by the FDOH and the University of South Florida Institutional Review Boards.

**Figure 1:**
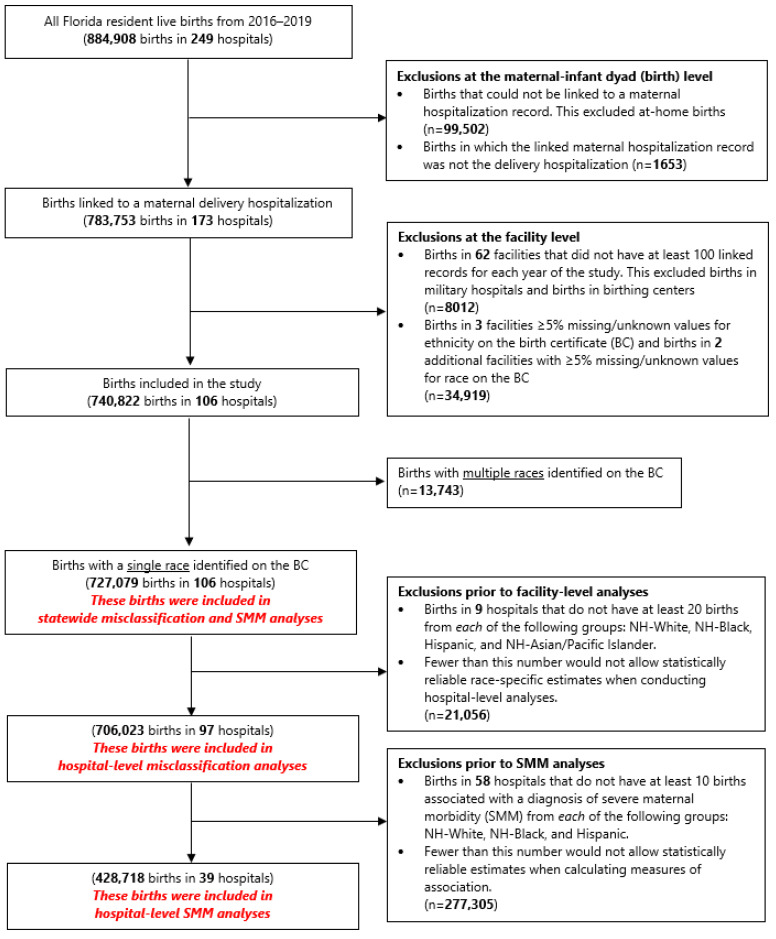
Flowchart describing determination of the study sample to be used in each analysis.

This study analysis consisted of three different subgroups, henceforth referred to as the state-level, hospital-level, and hospital-level SMM populations (Figure 1). The original study file of births from BC linked to maternal delivery hospitalizations was 783,753 births in 173 hospitals. For the state-level population, we excluded: (1) non-delivery hospitals, which are hospitals with less than 100 annual births; (2) military hospitals, birthing centers, and non-hospital births; and (3) facilities/hospitals with at least 5% missing or unknown values for the race or ethnicity variables on the BC to reduce potential bias (67 hospitals, *n* = 42,931). We also excluded births with mothers reporting multiracial heritage on the BC (*n* = 13,743) because multiracial heritage is not captured on the HD record. The study population at the state level included 727,079 births from 106 hospitals. Hospital-level and hospital-level SMM populations have further exclusions and are described later.

### 2.2. Measures

We extracted race and ethnicity information from both the BC and HD data sources. The HD record allowed the classification of race into categories (White, Black, American Indian or Alaska Native, Asian, Native Hawaiian or Pacific Islander, and other) (Table S1). Due to small individual sample sizes, we combined Asians and Native Hawaiian/Pacific Islanders into a single Asian-Pacific Islander group. On the BC, race can be defined across 15 different categories, which we recoded to align with the five race categories from the HD record. Although Hispanic subgroup differentiation is possible on the BC (e.g., Puerto Rican, Mexican), such granularity is not available on the HD record. As such, ethnicity was dichotomized as either Hispanic or non-Hispanic in the BC to align with the HD. Next, to reflect what is often performed for hospital reporting and in research studies, we created a combined race and ethnicity variable for analyses. All Hispanics were grouped together, and non-Hispanics were subclassified on the basis of reported race: non-Hispanic White (NH-White), non-Hispanic Black (NH-Black), non-Hispanic Asian Pacific Islander (NH-API), non-Hispanic American Indian or Alaska Native (NH-AIAN), and non-Hispanic “other”.

Since mothers’ self-reported race and ethnicity as documented on the BC, are typically based on US recommendations [25], the BC is used as the gold standard for all race and ethnicity comparisons in this study. Any classification of race and ethnicity reported on the HD that did not match the race and ethnicity reported on the BC was considered a misclassification. To reflect the impact of race and ethnicity misclassification on a meaningful perinatal outcome, we created a dichotomous indicator of SMM presence (yes or no) based on International Classification of Diseases, Tenth Edition (ICD-10) diagnosis and procedure codes documented on the delivery hospitalization record, and defined using the Alliance for Innovation on Maternal Health’s (AIM) ICD-10 criteria for 21 indicator conditions [26].

### 2.3. Analyses

We calculated the sensitivity, specificity, positive predictive value (PPV), and negative predictive value (NPV) separately for (1) race, (2) ethnicity, and (3) combined race and ethnicity variables to analyze state-level misclassification between the BC and HD using state-level population. Sensitivity estimates the probability that the HD captures race and ethnicity as reported in the BC. For example, we calculated the sensitivity of NH-AIAN as the proportion of all individuals reported as NH-AIAN on the BC that were correctly identified as NH-AIAN on the HD. PPV estimates how frequently the HD was accurate in classifying a specific race and ethnicity among those who reported as that race and ethnicity. For example, we calculated the PPV for NH-AIAN as the proportion of NH-AIAN on the HD that was classified as NH-AIAN on the BC. We made these calculations in two ways. First, we considered missing race and/or ethnicity in one data source and non-missing race and/or ethnicity in the other source as disagreement. Second, we excluded records with missing race and ethnicity (Table S2) and estimated these measures only among records with non-missing race and ethnicity in both data sources. The sensitivity analysis comparing race and ethnicity between BC and HD resulted in minimal differences with and without missing data. Therefore, analyses presented in this paper use data inclusive of missing race and ethnicity variables.

We then assessed the impact of maternal race and ethnicity misclassification on SMM at the state level by first calculating the number of SMM cases, SMM rates, and risk ratios representing the association between race and ethnicity and SMM using each data source (BC, HD) individually. We then calculated the percent difference in these SMM statistics when using race and ethnicity from the HD versus the BC data.

To further examine misclassification at the hospital level, we used a subset of the state-level population. The hospital-level population excluded nine additional hospitals that did not have at least 20 births from each race and ethnic group—NH-White, NH-Black, Hispanic, and NH-API—in order to ensure statistically reliable estimates for each race and ethnicity category when calculated at the hospital level. The hospital-level population consisted of 706,023 births from 97 hospitals. We analyzed the distribution (minimum, maximum, median, interquartile range) of sensitivity, specificity, PPV, and NPV measures across hospitals. To then assess how race and ethnicity misclassification affects SMM rate at the hospital level, we used a subset population of the hospital-level population, where we excluded an additional 58 hospitals that did not have at least 10 births with SMM from each of the following groups: NH-White, NH-Black, and Hispanic. Again, this was performed in order to have minimally reliable estimates of SMM rates at the hospital level; insufficient numbers of NH-API were available at the hospital level for inclusion. The hospital-level SMM population consisted of 428,718 births from 39 hospitals. We conducted a sensitivity analysis comparing the sensitivity and PPV findings for these 39 maternity hospitals to the overall 97 maternity hospitals in the state. For each race/ethnic group, we then examined the distribution of hospital-level percent differences in rates of SMM diagnosed in maternal delivery HD records compared to BCs.

All analyses for this study were conducted using SAS software version 9.4 (SAS Institute Inc, Cary, NC, USA), and figures were generated using R version 4.1.3 with RStudio 2022.02.3.

## 3. Results

**Race and Ethnicity.** At the state level, NH-AIAN was the most misclassified race and ethnicity (sensitivity: 28.2%, PPV: 25.2%) and was commonly misclassified as NH-API (30.3%) in HD records (see Table 1). NH-AIAN was most often incorrectly classified as another race than classified correctly as NH-AIAN. NH-API was the next most misclassified group (sensitivity: 57.3%, PPV: 85.4%) and was commonly misclassified as NH-White (5.8%) or non-Hispanic Other (5.5%). The next three groups represented the largest percentages of Florida births and were misclassified far less frequently. Most Hispanic mothers were reported as Hispanic (sensitivity: 80.8%, PPV: 91.0%) but were also reported as NH-White (6.3%). NH-White were also reported as such (sensitivity: 90.7%, PPV: 89.6%) but were also reported as Hispanic (7.7%); NH-Black (sensitivity: 91.8%, PPV: 95.7%) were more frequently reported accurately but were also misclassified as Hispanic (1.9%).

**Race and Ethnicity Separately.** The sensitivity and PPV for each race group were fairly similar to the findings of the combined category (see Table 1). As a separate category, Hispanic ethnicity (sensitivity: 80.8%, PPV: 91.0%) was more commonly misclassified than NH ethnicity (PPV: 93.2%; sensitivity: 94.6%).

### 3.1. Hospital Level

**Figure 2:**
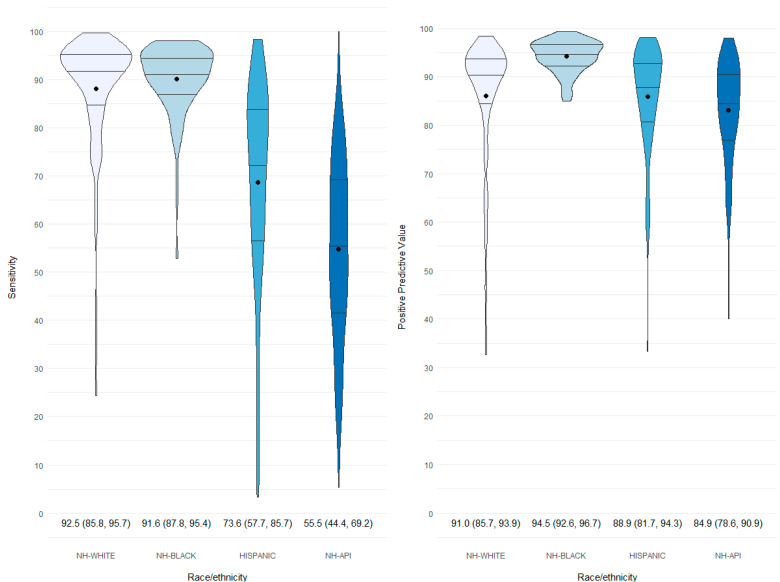
Distribution of hospital-level sensitivity (left) and positive predictive value (right) measures reflecting agreement of race and ethnicity documentation between birth certificates and maternal delivery hospitalization discharge records, 97 hospitals, Florida, 2016–2019. PPV, positive predictive value; NH, non-Hispanic; API, Asian or Pacific Islander; “●,” mean value; “–” mean quartiles (estimates presented below each plot). Note: A hospital must have at least 20 births in each race/ethnic group.

At the hospital level, wide variation in sensitivity and PPV with negative skewing was identified, particularly for NH-White, Hispanic, and NH-API groups (Table 2). The violin plots represented in Figure 2 show the distribution of sensitivity and PPV for race and ethnicity at the hospital level. Evidenced by higher medians (IQR) for sensitivity, more hospitals correctly classified NH-White 92.5 (85.8, 95.7) and NH-Black 91.6 (87.8, 95.4). Wider variation is seen across hospitals in correctly classifying Hispanic 73.6 (57.7, 85.7) and NH-API 55.5 (44.4, 69.2) race and ethnicity groups. The probability of being truly NH-Black when classified as NH-Black in HD records (PPV) was highest among all hospitals, 94.5 (92.6, 96.7).

### 3.2. Impact of Misclassification on a Quality Indicator (SMM)

**Figure 3:**
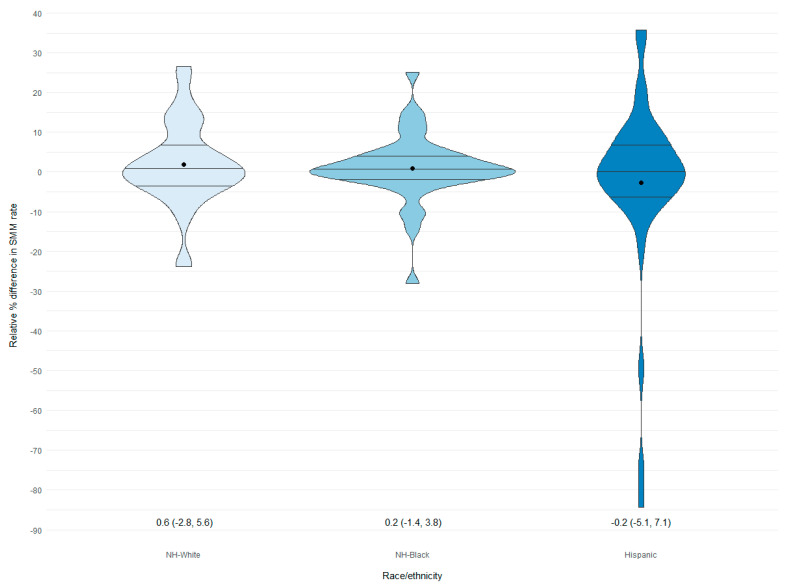
Distribution of hospital-level percent difference in race and ethnicity-specific rates of severe maternal morbidity diagnosed in maternal delivery hospitalization discharge records compared to birth certificates, 39 hospitals, Florida, 2016–2019. SMM, severe maternal morbidity NH, non-Hispanic; “●,” mean value; “–” mean quartiles (estimates presented below each plot). Note: A hospital must have had at least 10 SMM events in each race/ethnic group to be included. Percent difference = ((SMM rate HD - SMM rate BC)/SMM rate BC) × 100.

Generally, misclassification did not result in large SMM rate differences at the state level for most race and ethnicity categories (Table 2). However, as a result of misclassification, SMM rates for NH-AIAN appeared to be higher (% difference: 78.7) when using HD data. Although clear variability occurred among all three race and ethnicity groups at the hospital level, Hispanics had wide variability of the percent difference in SMM rates when using HD data compared to BC data and were more likely to have underestimated SMM rates (Figure 3).

## 4. Discussion

We found that misclassification at the state level varied amongst all racial and ethnic groups, i.e., NH-White, NH-Black, Hispanic, NH-API, and NH-AIAN groups. The most misclassified groups were NH-API and NH-AIAN women, and the least misclassified group was NH-Black women. A wide variation in misclassification for all races and ethnic groups was observed at the hospital level. Misclassification of race and ethnic groups resulted in differences in SMM rates for the NH-AIAN group at the state level and among all three groups at the hospital level, especially the Hispanic group.

At the state level, Florida’s sensitivity and PPV of HD data for race and ethnicity classification at delivery varies widely across subgroups. NH-API and NH-AIAN women were by far the most misclassified group; NH-Black women were the least misclassified. Howland et al. [27] is the only US-published study to investigate HD race and ethnicity reporting accuracy among maternity populations, where they too found sensitivity was highest among NH-Black women and lowest among NH-API and Hispanic women [27]. Unlike this New York state study, our study with a larger sample size showed wide variation in PPV across all race and ethnicity groups. Importantly, we included NH-AIAN women, who are understudied due to small sample sizes, and they were more likely to be classified incorrectly as NH-API or NH-White than as NH-AIAN. NH-AIAN individuals are the most misclassified group [14,17] and are more often classified as another group (i.e., NH-API). This is consistent with previous studies in non-maternity populations and utilizing different data sources [14,17].

Race and ethnicity misclassification did not result in major differences in SMM rates across NH-White, NH-Black, Hispanic, and NH-API women and is consistent with findings by Howland et al. [27]. However, state-level SMM rates for NH-AIAN were greatly impacted in our study. Although SMM rates of NH-White, NH-Black, Hispanic, and NH-API race and ethnicity groups may be accurate at the state level, Florida’s SMM rates for NH-AIAN deliveries are greatly underestimated. Similarly, several studies focused on NH-AIAN have shown that the misclassification of this group has also led to an underestimation of other health disparities [14,15,17,28,29,30].

At a hospital level, wide variation in sensitivity and PPV exists, resulting in frequently unreliable hospital data for race and ethnicity groups. Most hospitals accurately classified NH-Black and NH-White women, with a sensitivity of at least 85%. However, 25% and 50% of hospitals did not capture race and ethnicity correctly for Hispanics and NH-API, respectively. While classification at the state level for NH-White, NH-Black, and Hispanics was mostly accurate, the extent of race and ethnic misclassification across hospitals is inconsistent, making it challenging to predict the impact on outcome rates for a given hospital.

Wide accuracy variation in hospital sensitivity and PPV can be misleading and result in considerable underestimation of SMM rates for some race and ethnic groups, especially Hispanics. Despite our ability to assess the impact of NH-API and NH-AIAN misclassification on SMM rates at a state level, we anticipate that these two groups would be more greatly impacted at a hospital level. Our findings provide one of the first looks at the hospital level on the prevalence of racial and ethnic misclassification among maternity patients overall and the impact on maternal outcomes.

### 4.1. Clinical Implications

Our study demonstrated wide variability of race and ethnicity misclassification in HD records at the state level for all race and ethnic groups, especially for the NH-API and NH-AIAN groups, and wide variability at the hospital level for NH-White, Hispanic, and NH-API groups. Misclassification at the state level did not result in large SMM rate differences, except for the NH-AIAN group, where we observed an overestimation of SMM rates. At the hospital level, misclassification appeared to result in an underestimation of SMM rates for all three groups but especially the Hispanic group. This misclassification likely impacts multiple health and health quality measures at a hospital level. Therefore, health agencies and hospitals should not rely on state-level race and ethnicity data as a proxy for assessing accuracy at the hospital level. This study highlights the need for uniform processes in collecting self-reported race and ethnicity, nativity, and country of origin, and patient education on the importance of ascertaining this information in order to improve their health care. Importantly, correct classification of race and ethnicity would potentially ensure that the delivery of health care services is tailored more appropriately.

### 4.2. Research Implications

Health differences stemming from race and ethnicity are a health priority because they can be used to assess unmeasured social and health factors that influence health and health care delivery. To obtain a true measure of outcome differences, data sources used to identify race and ethnicity must be accurate and reliable. In the US, reducing race and ethnicity misclassification in HD data is key to monitoring and reducing SMM and other maternal health outcomes at the state- and hospital level, where improvements can be made. Future studies are needed to further delineate the implications of misclassification among people of multiracial heritage on health outcomes at a state and hospital level.

### 4.3. Strengths and Limitations

One strength of the study is Florida’s large, highly diverse birthing population, which provides a large sample size for population subgroups. This enabled assessing smaller race and ethnicity categories such as NH-AIAN and NH-API. Second, it has become a priority to obtain accurate estimates of race and ethnicity data at the state-, county-, and hospital levels because of the increasing diversity across many US states and in an effort to monitor and address disparities [31]. Therefore, although race and ethnicity make up varies from state to state, our study is applicable across all US states. Third, despite HD data quality issues, we were able to examine the effect of misclassification on SMM, a major national health care quality indicator. Fourth, this study not only assessed misclassification at a state level but evaluated these key measures across hospitals [27]. Our study also has some limitations. The reporting sources for race and ethnicity data collection on hospital discharge are unclear and likely vary across hospitals. Some racial and ethnic groups were of small size in Florida, limiting the scope of analyses. In addition, HD categories were limiting. Using the linked file in Florida excluded certain groups, especially undocumented women. This may have only a small impact [32]. The generalizability of our hospital-level findings is somewhat limited to large US maternity hospitals. However, state-level sensitivity and PPV findings for large maternity hospitals in Florida were very similar to overall state-level findings on sensitivity analysis (Figure S1). Our findings are not generalizable to other countries. The Organisation for Economic Co-operation and Development (OECD, Paris, France) outlines the data collection practices of racial, ethnic, and indigenous identities in member countries [33]. Some countries do not collect race and ethnicity data [34]. For example, in Denmark, the country of origin was utilized as a proxy for ethnicity in one study [35]. Furthermore, race and ethnic groups may differ from the US. For example, in Brazil, race and ethnicity are defined as White, Pardo (the official term for the admixed population), Black, Asian, and Indigenous [36]. Likewise, in the United Kingdom, where greater than 10 racial ethnic groups exist, one study assessed misclassification with 16 different race and ethnicity groups [37]. However, other countries may find similar misclassification of disparate groups, and our study may prompt them to conduct such investigations to accurately assess maternity health outcomes [35,36,38,39].

## 5. Conclusions

This study assessed the differences in race and ethnicity reporting between HD and BC data from maternity hospitals in Florida and estimated the impact of race and ethnicity misclassification on state- and hospital-specific SMM rates. Misclassification of race and ethnicity in HD records occurs at both the state and hospital levels, resulting in differences in SMM rates at each level. The role of race and ethnicity may be different in other countries. Reducing race and ethnicity misclassification is key in assessing and addressing SMM rate differences and better informing surveillance, research, and quality improvement efforts.

## Figures and Tables

**Table 1:** Comparing documentation of maternal race and ethnicity on birth certificates compared to the maternal delivery hospitalization discharge record, Florida, 2016–2019.

Variable	Category	*n*	Sensitivity	Specificity	PPV	NPV
Race and ethnicity	NH-White	321,677	90.7	91.6	89.6	92.5
	NH-Black	163,591	91.8	98.8	95.7	97.6
	Hispanic	207,817	80.8	96.8	91.0	92.6
	NH-API	23,382	57.3	99.7	85.4	98.6
	NH-AIAN	805	28.2	99.9	25.2	99.9
Race	White	521,665	84.8	93.6	97.1	70.8
	Black	169,071	93.1	98.6	95.3	97.9
	API	23,842	59.1	99.6	84.3	98.6
	AIAN	908	28.3	99.8	17.4	99.9
Ethnicity	Hispanic	207,817	80.8	96.8	91.0	92.6
	Non-Hispanic	516,544	94.6	83.1	93.2	86.1

PPV, positive predictive value; NPV, negative predictive value; NH, non-Hispanic; API, Asian or Pacific Islander; AIAN, American Indian or Alaskan Native. Note: Missing values are included as disagreement.

**Table 2:** Statewide cases and rate of severe maternal morbidity diagnosed in maternal delivery hospitalization discharge records compared to birth certificates, by race and ethnicity, Florida, 2016–2019.

Race/Ethnic Group	Hospital Discharge	Birth Certificates	% Difference
**NH-White**			
Live births	325,548	321,677	1.2
SMM cases	4091	3977	2.9
SMM risk (%)	1.3 (1.2, 1.3)	1.2 (1.2, 1.3)	1.7
SMM risk ratio	reference	reference	n/a
**NH-Black**			
Live births	156,879	163,591	−4.1
SMM cases	3611	3680	−1.9
SMM risk (%)	2.3 (2.2, 2.4)	2.2 (2.2, 2.3)	2.3
SMM risk ratio	1.83 (1.75, 1.91)	1.82 (1.74, 1.90)	0.7
**Hispanic**			
Live births	184,558	207,817	−11.2
SMM cases	2742	3051	−10.1
SMM risk (%)	1.5 (1.4, 1.5)	1.5 (1.4, 1.5)	1.2
SMM risk ratio	1.18 (1.13, 1.24)	1.19 (1.13, 1.24)	−0.4
**NH-API**			
Live births	15,676	23,382	−33.0
SMM cases	254	391	−35.0
SMM risk (%)	1.6 (1.4, 1.8)	1.7 (1.5, 1.8)	−3.1
SMM risk ratio	1.29 (1.14, 1.46)	1.35 (1.22, 1.50)	−4.7
**NH-AIAN**			
Live births	901	805	11.9
SMM cases	20	10	100.0
SMM risk (%)	2.2 (1.3, 3.2)	1.2 (0.5, 2.0)	78.7
SMM risk ratio	1.77 (1.14, 2.73)	1.00 (0.54, 1.86)	75.8
**NH-Other**			
Live births	21,424	6627	223.3
SMM cases	291	92	216.3
SMM risk (%)	1.4 (1.2, 1.5)	1.4 (1.1, 1.7)	−2.2
SMM risk ratio	1.08 (0.96, 1.22)	1.12 (0.91, 1.38)	−3.7

NH, non-Hispanic; API, Asian or Pacific Islander; AIAN, American Indian or Alaskan NativeNote: SMM risk ratio compares the risk of SMM for each race/ethnic group compared to NH-White. The percent difference is calculated as: ((hospital discharge measure−birth certificate measure)/birth certificate measure) × 100.

## Data Availability

Restrictions apply to the availability of these data. Data were obtained from the Florida Department of Health and the Florida Agency for Health Care Administration.

## Supplementary Materials

The following supporting information can be downloaded at: https://www.mdpi.com/article/10.3390/ijerph20095689/s1, Figure S1: Comparing distribution of hospital-level sensitivity (top) and positive predictive value (bottom) measures, by race and ethnicity, between the original 97 hospitals and the subset of 39 hospitals included in analysis of severe maternal morbidity, Florida, 2016–2019; Table S1: Classification of race groups in BC records to match the race groups provided in HD records; Table S2: Comparing documentation of maternal race and ethnicity on birth certificates compared to the maternal delivery hospitalization discharge record, Florida, 2016–2019.
