# Supplementary material for: Race and Ethnicity Misclassification in Hospital Discharge Data and the Impact on Differences in Severe Maternal Morbidity Rates in Florida
Source: Int J Environ Res Public Health. 2023 Apr 30;20(9):5689. doi: 10.3390/ijerph20095689 (PMC10178402; doi:10.3390/ijerph20095689)

Table S1. Classification of race groups in BC records to match the race groups provided in HD records

Race in BC	Race in HD
White	White
Black	Black
American Indian	American Indian or Alaskan Native
Chinese	Asian
Japanese	Asian
Vietnamese	Asian
Korean	Asian
Filipino	Asian
Indian	Asian
Other Asian	Asian
Hawaiian	Asian Pacific Islander
Guam	Asian Pacific Islander
Samoan	Asian Pacific Islander
Pacific Islander	Asian Pacific Islander
Other	Other
Unknown	Unknown

Table S2. Comparing documentation of maternal race and ethnicity on birth certificates compared to the maternal delivery hospitalization discharge record, Florida, 2016–2019

Variable	Missing Values [a]	Category	N	Sensitivity	Specificity	PPV	NPV
Race-ethnicity	Included	NH-White	321,677	90.7	91.6	89.6	92.5
		NH-Black	163,591	91.8	98.8	95.7	97.6
		Hispanic	207,817	80.8	96.8	91.0	92.6
		NH-API	23,382	57.3	99.7	85.4	98.6
		NH-AIAN	805	28.2	99.9	25.2	99.9
	Excluded	NH-White	312,437	93.3	91.6	90.0	94.4
		NH-Black	159,194	94.3	98.8	96.0	98.3
		Hispanic	198,433	83.3	96.9	91.4	93.6
		NH-API	21,843	61.3	99.7	85.9	98.8
		NH-AIAN	754	30.1	99.9	25.6	99.9
Race	Included	White	521,665	84.8	93.6	97.1	70.8
		Black	169,071	93.1	98.6	95.3	97.9
		API	23,842	59.1	99.6	84.3	98.6
		AIAN	908	28.3	99.8	17.4	99.9
	Excluded	White	502,938	86.4	94.0	97.4	73.0
		Black	164,099	94.1	98.6	95.5	98.2
		API	22,239	61.2	99.6	84.6	98.7
		AIAN	849	28.5	99.8	17.1	99.9
Ethnicity	Included	Hispanic	207,817	80.8	96.8	91.0	92.6
		Non-Hispanic	516,544	94.6	83.1	93.2	86.1
	Excluded	Hispanic	198,433	83.3	96.9	91.4	93.6
		Non-Hispanic	500,467	96.9	83.3	93.6	91.4

PPV, positive predictive value; NPV, negative predictive value; NH, non-Hispanic; API, Asian or Pacific Islander; AIAN, American Indian or Alaskan Native

[a] When missing values are excluded, disagreement only occurs when different non-missing categories are assigned. When missing values are included, disagreement can occur when one data source is missing, regardless of the category assigned by the other data source.

Figure S1. Comparing distribution of hospital-level sensitivity (top) and positive predictive value (bottom) measures, by race and ethnicity, between the original 97 hospitals and the subset of 39 hospitals included in analysis of severe maternal morbidity, Florida, 2016–2019

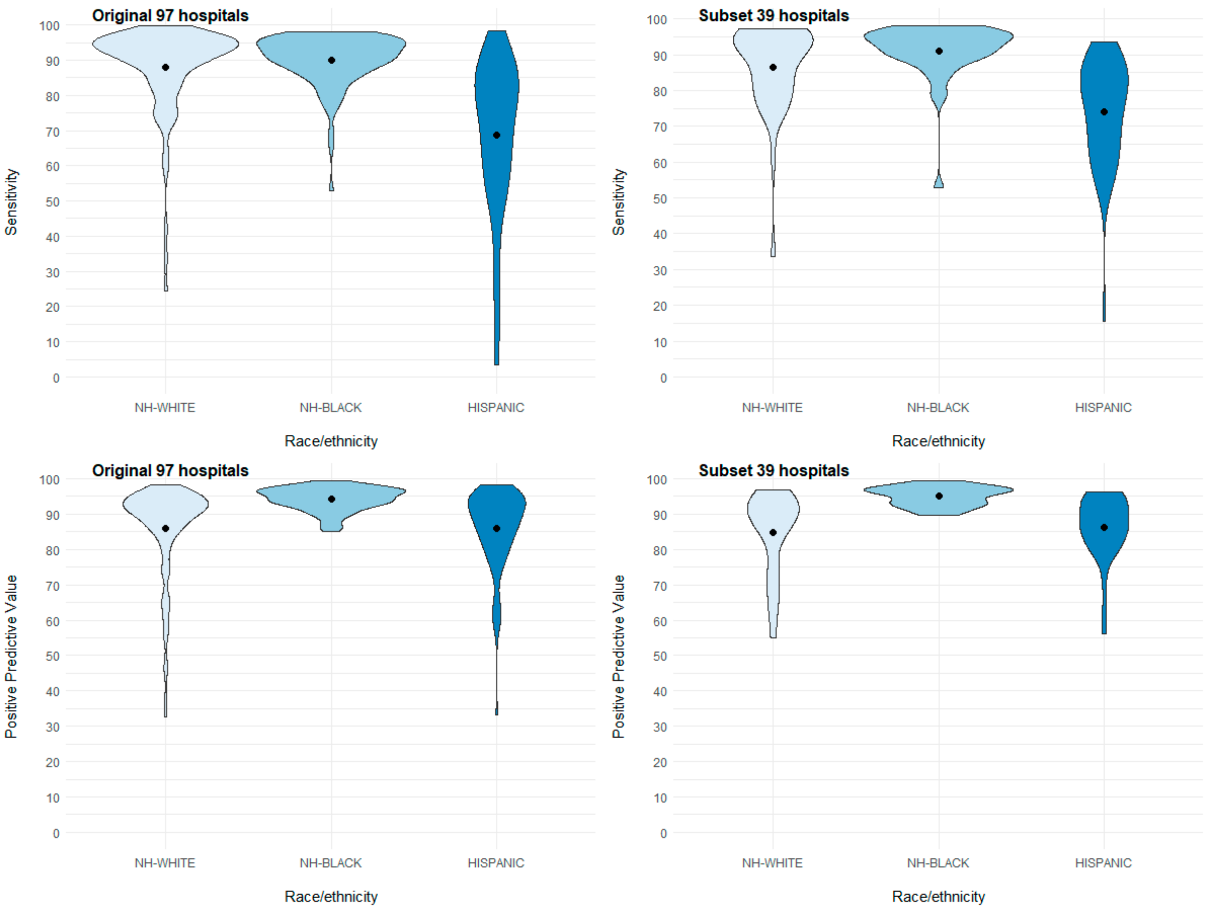

Supplement: Supplementary file 1 [file ijerph-20-05689-s001.zip › ijerph-2192024-supplementary.pdf]
